# Relationships between intrinsic population growth rate, carrying capacity and metabolism in microbial populations

**DOI:** 10.1038/s41396-023-01543-5

**Published:** 2023-10-27

**Authors:** Dustin J. Marshall, Hayley E. Cameron, Michel Loreau

**Affiliations:** 1 School of Biological Sciences/Centre for Geometric Biology, Monash University, Victoria, 3800 VIC Australia; 2 Theoretical and Experimental Ecology Station, CNRS, 2 route du CNRS, 09200 Moulis, France

**Keywords:** Microbial ecology, Population dynamics

## Introduction

In their simplest form, the dynamics of populations are described in terms of two parameters: *r*, the intrinsic rate of increase; and *K*, the carrying capacity of the population. These two parameters are fundamental to population ecology and have a long history of empirical and theoretical study [1]. From an evolutionary perspective, *r* and *K* were used to define and describe different modes of life: *r*-strategists were thought to have fast population growth rates at the expense of poor competitive abilities; *K*-strategists were thought to have slow-growing populations but be superior competitors, or at least more efficient with regards to resources [2]. These concepts have strongly influenced microbial ecologists and evolutionary biologists—*r* and *K* are often expected to trade off against each other across genotypes, strains or species [3]. Since then, the classification of *r*- and *K*-strategists has been adapted by microbiologists to describe copiotrophic and oligotrophic species of microorganisms, respectively [4]. The idea that it is difficult to have both fast growth and be efficient in the use of resources has intuitive appeal: multiple mechanistic models attempt to explain how and why we might observe trade-offs between *r* and *K* [5, 6]. However, empirical studies struggle to detect trade-offs between *r* and *K* at multiple levels of biological organisation and instead sometimes even detect ‘trade-ups’ where *r* and *K* positively covary [7, 8]. Even within the same microbial strains, different *r*-*K* relationships can be observed depending on environmental quality [8]. Similarly, comparisons across species fail to reveal simple oligotrophy and copiotrophy (or r-*K* strategist) dichotomies—instead species often fall on a continuum between these two extremes [9]. In fact, expectations about how *r* and *K* covary with each other are based on an unfortunate quirk of scientific fate.

The intrinsic rate of increase, *r*, and the carrying capacity of populations, *K*, are much more related than most people realise. Jim Mallet provides a useful history of this issue and we refer readers to his elegant exposition for more detail [1]. Briefly, the most common formulation of the logistic equation for population growth, which includes the familiar *r* and *K*, came about because one influential text published a century ago used that particular formulation, where *per capita* population growth rate (*R*) is given by Eq. (1):

1 $$R=r\left(1-\,\frac{N}{K}\right)$$

Where *r* is the intrinsic rate of growth, *K* is carrying capacity and *N* is population size. This equation, plus observations that species with high values of *r* tend to have lower values of *K*, led to the idea that *r* and *K* are biological parameters that show covariance but are also ‘separate’ from each other in a mathematical sense. However, as multiple authors have pointed out over time [1, 3, 10], the original formulation of the logistic equation was more focused on the biological processes rather than the outcomes of those processes, having the form of Eq. (2):

2 $$R=r-\alpha N$$

Where *α* can be interpreted as the equivalent of the intraspecific competition coefficient. Equations (1) and (2) are actually equivalent: when the *per capita* growth rate *R* = 0, *N*’ will equal *r*/α, equivalent to *K* in Eq. (1). The essential difference between these formulations, however, is that in Eq. (1) *K* appears to be an independent biological parameter defined by the organism’s traits and the environment, whereas *N*’ is an outcome of the rate at which the population grows and the intensity of the density-dependence that the population experiences. While both equations describe the exact same dynamics, Eq. (2) emphasises the underlying biological processes of density dependence and population growth rates, whereas Eq. (1) emphasises the endpoints of those processes at equilibrium. As Mallet points out, Eq. (2) is also more readily applied to matter and energy fluxes, and provides a more natural bridge between ecological and evolutionary models [1]. For our purposes here, however, Eq. (2) makes a crucial issue clear, that any consideration of the equilibrium population density, known as *K*, is not independent from *r*. For clarity, here is the function that relates *r* to *K* at equilibrium:

3 $$K=\frac{r}{\alpha }$$

Equation (3) implies that our expectations for *r*–*K* relationships in microbial ecology, and biology more generally, have been misplaced. A deeper exploration of Eq. (3) and its components reveals fascinating biological implications of different *r*–*K* relationships. We will deal with each of these issues in turn.

## Should we expect a trade-off between *r* and *K*?

Despite longstanding discussions about different life history traits, such as: resistance and tolerance; *r*–*K* strategies; resource-use efficiency etc., Eq. (3) demonstrates that we have no reason to expect a negative relationship between *r* and *K*. In fact, all else being equal, *K* should be *proportional* to *r*—simply put, because *K* contains *r*, *ceteris paribus*, anything that increases *r* will also increase *K* at the same rate. While not broadly appreciated, MacArthur [10] provides an excellent mechanistic derivation that predicts that *r* and *K* should covary positively and proportionately to each other.

From this perspective, ‘trade-ups’ between *r* and *K* are not unexpected but instead, might be regarded as the default expectation, and trade-offs should instead be considered a special case. If the relationship between *r* and *α* is positive and linear, then *r* and *K* will show no covariance. Indeed, *r* and *K* can only covary negatively under a very specific condition: when α increases with *r* nonlinearly; specifically, with the form that the scaling exponent linking *r* to *α* is greater than 1. In more biological terms, *r*-*K* trade-offs will only be observed when slight increases in *r* yield disproportionate increases in the strength of density dependence. Later, we will explore some situations when disproportionate relationships between *r* and density-dependence might be expected.

Equation (3) illustrates that *r* and *K* can show any relationship, from negative to positive. The failure to observe a negative relationship between *r* and *K* across any number of studies, therefore, transitions from being a paradox requiring a special explanation into a more natural reflection of the relationship between these two parameters. Our argument here is not that *K* is worthless or should not be estimated, instead we want to emphasise that *K* is a useful description of the outcome of two biological processes (intrinsic growth and density-dependence), instead of being some kind of independent biological parameter in its own right.

Equation (3) also highlights the fact that discussions over which population parameter is maximised by evolution, be it *r* or *K* [11], are somewhat misplaced. Because *K* contains *r*, evolution could maximise both simultaneously, if *α* is left unaffected, simply by acting to increase *r* on its own. Likewise, evolution in *r* might actually be entirely due to selection acting on *K*—this counterintuitive example illustrates the intrinsic connection between the two parameters. Given that *r* and *K* are so tightly bound, why then do we observe very different relationships between the two from study to study, organism to organism or even environment to environment? To answer this question, we need to first decompose *r*, and *α* into their underlying constituents and access the biology they represent.

## What biology is in *r* and *α*?

The intrinsic rate of increase, *r*, is the difference between the birth rate (*b*_*0*_) and the death rate (*d*_*0*_) without any effect of density (this is not strictly true, but will serve for our purposes) such that:

4 $$r={b}_{0}-{d}_{0}$$

For microbial organisms, then, anything that increases the rate at which cells divide, or decreases the rate that cells die (or both) will increase *r*. Meanwhile, α is given by:

5 $$\alpha =-(\beta -\delta )$$

where *β* is the density-dependence of birth rates (cell division rates), which is assumed to linearly decline with population density so *β* < 0, and *δ* is the density-dependence of death rates, which is assumed to linearly increase with density so *δ* > 0. The sum of *β* and −*δ* will typically be negative. To put these coefficients into a biological context, imagine a population where *r* = 3. If *β* = −0.05 and *δ* = 0.01, that would mean that if ten cells were added to the population, the *per capita* growth rate (*R*) would drop to 2.4 [i.e. *R* = 3−((−(−0.05−0.01) × 10)]. Anything that makes the decline in birth rates with density steeper, or makes the relationship between deaths and density more positive, will increase *α*, and so decrease *K*.

We can see that a population’s equilibrium density or carrying capacity (*K*) emerges from the difference between density-independent rates of birth and death (*r*) and the density-dependence of those rates (*α*). Now we can start to explore different scenarios under which we might observe different relationships between *r* and *K*.

## Why might *r* and *K* covary positively?

First, *r* and *K* will positively covary (or ‘trade-up’) if *r* increases with no change in α. For example, imagine *E. coli* is placed into an environment that contains an antibiotic (e.g. 7). Let’s imagine the antibiotic only affects the mortality rate of cells per unit time, such that *d*_*0*_ increases: we will therefore observe a drop in *r* and, because nothing else is affected, we will also observe a drop in *K* (as per Eq. (3)). Let’s then imagine that the population evolves resistance to the antibiotic such that *d*_*0*_ decreases and nothing else changes. We will then observe an increase in *r* (and hence *K*) as the antibiotic resistance evolves. Comparing *r* and *K* of our focal population at different time points will therefore reveal a positive relationship between the two parameters. Indeed, that is exactly what is observed in studies of yeast and *E. coli* that adapt to harsh environments—both *r* and *K* increase [7, 8]. In the context of Eq. (3), these empirical results seem straightforward, but in both cases, the authors of the studies invoked new mechanistic models to explain the findings. Instead, we suspect that these patterns occur because *r* evolved without any meaningful change in α and standard population dynamics unfolded according to Eq. (3). We further predict that many of the trade-ups between *r* and *K* that are observed when microorganisms adapt to harsh environments occur via this process, although this remains untested (estimates of cell mortality rates would provide key insights here). At the very least, our point is that once the relationship between *r* and *K* is better appreciated, positive relationships between these two population parameters are no longer surprising— but rather they are to be expected.

## Why might *r* and *K* covary negatively?

Let us now consider the other extreme, whereby a negative relationship between *r* and *K* is observed. Given Eq. (3), we have already noted that the only way in which *r* and *K* can covary negatively is if a slight increase in *r* yields a disproportionately large increase in *α*—why might that happen?

We suspect that evolutionary changes in energy metabolism generate negative relationships between *r* and *K* [3, 5, 12]. Imagine a microorganism is placed into a resource-rich environment where a high *r* is favoured. According to Eq. (5), organisms can increase *r* by increasing rates of cell division. If we assume that cells do evolve in different sizes or composition (not always a reasonable assumption, see [13]), then the only way to increase rates of cell division is to increase metabolic rates in order to power a higher rate of biological work. So, let’s imagine *r* increases linearly with *per capita* metabolic rates via changes in *b*_*0*_. Such metabolic rates will certainly increase *r* [14]. But changes in metabolism will also affect density dependence (*α*). Specifically, a previously unrecognised prediction of metabolic theory is that α is proportional to the square of metabolic rate [15]. In biological terms, this multiplicative effect of metabolism on α occurs because increasing metabolism increases the *per capita* rate of resource removal from the environment while simultaneously increasing the resource requirements of each individual in that environment. This “double whammy” effect means that competitive interactions intensify strongly (according to a square function) with slight increases in metabolism.

**Fig. 1:**
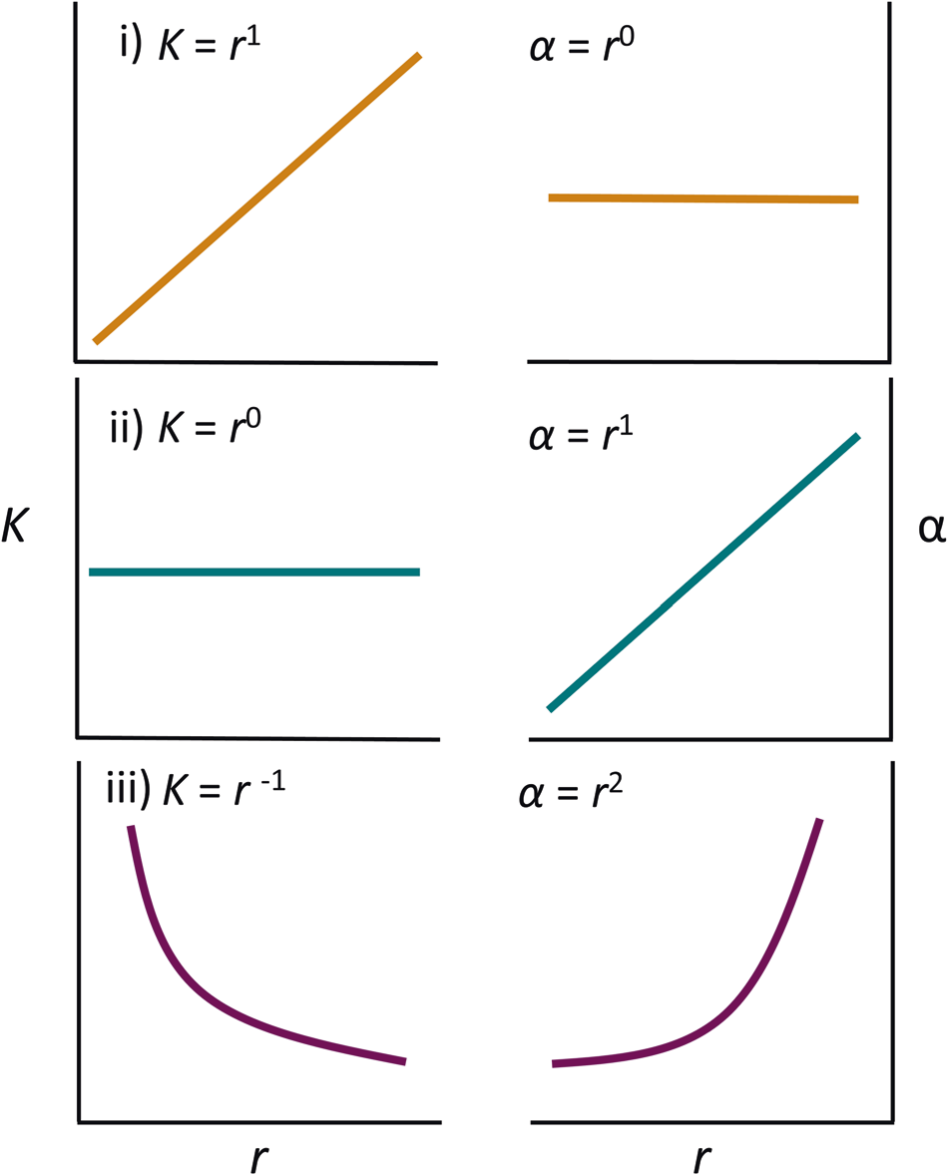
Schematic of the potential relationships between r, K and α. Scenario i): Increases in *r* have no effect on density dependence (*α*) so *r* and *K* covary positively (i.e. trade-up; e.g. the evolution of resistance increases *r*); Scenario ii): Increases in *r* increase density dependence (*α*) linearly so *r* and *K* show no relationship; Scenario iii): Increases in r cause nonlinear increases in density dependence (*α*) so *r* and *K* covary negatively (i.e. trade-off; e.g. the evolution of higher metabolic rates increase *r*).

From these equations, any small increase in metabolic rate that increases *r* yields a large increase in *α*. Putting these components together then, we predict that increases in *r* that come about solely via changes in *per capita* metabolic rate will yield concomitant changes in *K* such that:

6 $$r=a.\frac{1}{K}$$

where *a* is just some normalisation constant. In other words, *r* and *K* will negatively covary with each other, and show a scaling relationship of −1, if changes in metabolism drive changes in *r* and *K*. Studies of phytoplankton and the Long Term Evolution Experiment in *E. coli* show that metabolism can be a key mediator in the evolution of *r* and *K* across different resource regimes [13, 14]. We predict that the evolution of metabolic rate underpins the negative covariance between *r* and *K* but this requires further testing. We should also note that metabolic evolution is not the only way in which a negative covariance between *r* and *K* will be generated—anything that generates a relationship between *r* and α that scales at greater than 1 will cause the same pattern. For example, changes in cell size could have similar effects [13, 14]. For completeness, we should also note that if *r* and α covary positively but proportionately (i.e. classic arguments about efficiency versus growth rate often invoke such patterns; [5]), then *r* and *K* will actually show no covariance. In other words, even if increases in *r* generate increases in the strength of density dependence, unless these are nonlinear, then they will be insufficient to generate a classic *r*-*K* trade-off. We summarise the various possible relationships between *r*, *K* and *α* in Fig. 1.

## Extracting information from the diversity of *r* and *K* relationships

While we have focused on two interesting cases, whereby *r* evolves independently of *α*, or strongly in tandem with *α*, in reality these cases lie at either end of a continuum, and all possible combinations can be expected. This diversity of relationships between *r* and *K* provides more information about the underlying processes involved than has been appreciated. Specifically, the scaling of *r* and *K* provides deep insights into how rates of cell division (or births in more general terms) and death rates vary relative to the density-dependence of these rates (Fig. 1). When *r* and *K* positively covary, this implies that *r* has evolved in ways that are trivial to density dependence and any increases in *K* are simply because they were ‘dragged along’ by increases in *r* as per Eq. (3). The stronger the positive covariance between *r* and *K*, the less density-dependence was affected. In contrast, when *r* and *K* strongly covary negatively, any evolution of *r* has come with a massive concomitant change in the strength of density dependence. When *r* scales with *K* at or around −1, density dependence has increased with *r* steeply and nonlinearly (possibly through metabolic changes). In this light, the relationships between *r* and *K* seem far more informative and predictable.

**Fig. 2:**
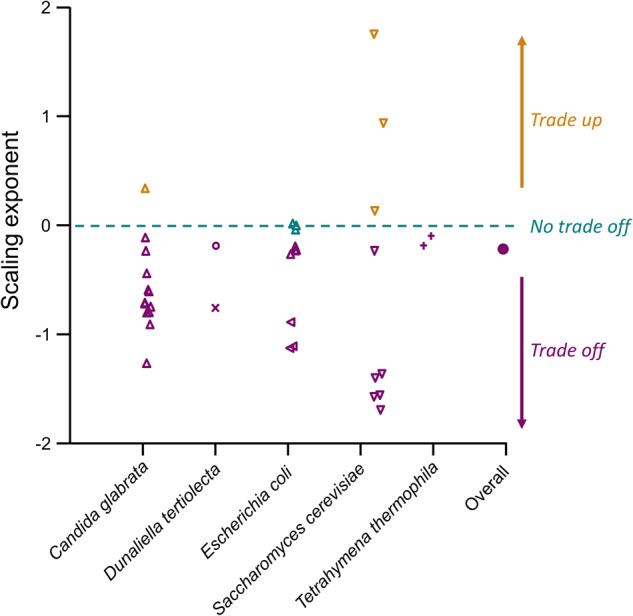
Estimates of the scaling relationship between *r* and *K* from our meta-analysis. Each point represents the scaling relationship for a given environment investigated in each study. Points indicate scaling exponents that are significantly negative (i.e. trade-off shown in purple), positive (i.e. trade-up shown in yellow), or are not significantly different from zero (i.e. no trade-off shown in blue). Different species investigated in these studies are shown on the *x* axis and symbols denote each study: Δ = [7], ○ = [14], ×= [16], ◁ = [17], ∇ = [8], + = [13]). The closed circle (●) indicates the central tendency of the scaling relationship based on all of the data.

We can use this new framework for interpreting *r*–*K* scaling relationships to make inferences about how microbe population dynamics evolve, specifically with regards to energy fluxes. We used a meta-analysis to examine how *r* and *K* scale with each other within any one environment for a range of microbes (see Supplementary Materials for detailed methods). We focused on studies where different strains, evolved lineages or genotypes of the same species were used to compare a range of *r* and *K* estimates, from which we could extract their scaling relationship. We did not include cross-species comparisons because among-species patterns do not describe within-species processes—which was our primary interest. We found six studies across 5 species and 36 different environments, that provided over 69,000 separate estimates of *r* and *K*. When we estimated the scaling relationships between *r* and *K* (Fig. 2) we find that most studies (29 of 36) find a negative scaling relationship; 3 find no relationship; and 4 find a positive scaling relationship. The central tendency across all studies is for a significantly negative scaling relationship (global estimate of *r*-*K* scaling [±C.I]: −0.233; [−0.280, −0.185]; *F*_1,65560_ = 92.7, *P* < 0.001), although the relationship between *r* and *K* depended strongly on the environment (environment interaction: *F*_34_, _65560_ = 489.5; *P* < 0.001). According to our framework, these findings indicate that evolution in *r* comes about most commonly via changes in energy requirements, either through changes in cell size or cell metabolism or both. Populations with higher intrinsic rates of growth achieve this via increased metabolic rates, which increases the strength of competitive interactions. Supporting this inference, of the three studies in our meta-analysis that estimated energy use [13, 14], all showed positive covariances between metabolic demands and *r*. It would be interesting to test metabolic rates in the other studies, those that have evolved negative covariances between *r* and *K* should show also changes in *per capita* energy demands.

Nevertheless, not all scaling relationships were negative, and some were strongly positive. Our framework holds that these positive covariances between *r* and *K* arose via differences in death rates rather than energy use—evolving a higher *r* through lower mortality rates should yield a higher *r* when *per capita* resource demands remain unaffected. Estimates of cell mortality rates would be useful for resolving this issue.

In one environment, we observed a scaling of ~1.7, far higher than might be expected if increases in *K* were solely driven by *r*. This high scaling value implies that genotypes with high *r* also evolved to have lower *α*—that genotypes that grew faster also experienced less density dependence. While the precise mechanism for this result is unclear, it is interesting that this result occurred in the only environment where an alternative carbon (galactose as opposed to glucose) source was provided. Our framework would imply genotypes that grew well on this carbon source were also more efficient in its use.

This preliminary meta-analysis illustrates that far more information about the demographic processes and energy fluxes that drive changes in *r-K* can be extracted by estimating how they scale with each other. For now, it seems that evolution in *r* often comes at the expense of *K* because of changes in energy metabolism but we eagerly await further tests of this hypothesis. At a broader evolutionary scale, we would argue that, as much as copiotrophs and oligotrophs are synonymous with classic *r*-*K* classifications, we should be characterising them in terms of the relative ratio of *r* and *α*, rather than based on values of *r* and *K*. As Mallet has also argued [1], doing so may facilitate better integration of the eco-evolutionary dynamics that yielded these patterns in the first place.

### Supplementary information

Supplementary Methods

Dataset 1
