# Supplementary material for: Relationships between intrinsic population growth rate, carrying capacity and metabolism in microbial populations
Source: ISME J. 2023 Oct 27;17(12):2140–3. doi: 10.1038/s41396-023-01543-5 (PMC10689727; doi:10.1038/s41396-023-01543-5)
Supplement: Supplementary file 1 — Supplementary Methods [file 41396_2023_1543_MOESM1_ESM.docx]

**Meta-analysis methods**

We searched Google Scholar on September 1^st^ 2023 using the search terms: "intrinsic rate" and "carrying capacity" and microb* evol*. To be included in our analysis, studies had to include estimates of *r* and *K* for a given environment and involve at least 3 different genotypes/lineages/strains of the same species evaluated in those environments. We excluded any studies that only estimated *r* and *K* for a single population or for replicates of the same population/strain/genotype/lineage. We got 12060 hits and scanned the first 400 results and found 5 studies that matched our criteria. All of the studies that were ultimately included in our analyses appeared in the first 100 records.

To analyse the scaling relationship between *r* and *K* within each environment, we extracted the raw data from online repositories for these studies and analysed the relationships directly using log-log linear analyses for each environment-species combination (Supplementary Table 1). To analyse the central tendency of the data overall, we fit a mixed-effects linear model that included *r* as a continuous predictor, environment as a categorical fixed effect, the interaction between r and environment, as well as the random effect of species and species nested with study.
